# A Qualitative Study of Healthcare Experience Among Chinese Homebound Adults Receiving Home-Based Medical Care

**DOI:** 10.1093/geroni/igaa057.157

**Published:** 2020-12-16

**Authors:** Rui Zhou, Joyce Cheng, Shuangshuang Wang, Nengliang (Aaron) Yao

**Affiliations:** 1 Shandong Uniiversity, Jinan, China; 2 University of Virginia, Springfield, Virginia, United States; 3 Shandong University, Jinan, Shandong, China

## Abstract

Home-based medical care (HBMC) is emerging in China, but research understanding the efficiency and effectiveness of this new care model is rare. In this study, researchers interviewed 17 Chinese homebound adults aged 45 and older (53% females, mean age=76) who have received HBMC, and collected detailed information regarding their experiences and attitudes toward HBMC. Participants were recruited from healthcare institutions in Shanghai, Jinan, and Zhangqiu of China. The evaluation of patients’ experiences with HBMC yielded both positive and negative aspects. Positive experiences included 1) the delivery method was convenient for homebound patients; 2) health problems could be detected timely because doctors visited patients regularly; 3) home care providers had better bedside manners and professional skills than hospital-based providers; 4) the medical insurance covered the cost of home care services. Negative experiences related to the supply and quality of care, including 1) the scope of current HBMC services was too limited to meet the needs of homebound patients; 2) the visit time was too short; 3) healthcare providers’ professional skills varied greatly. Findings from this study suggest that the HBMC model benefited Chinese older adults, primarily homebound adults, in terms of convenience and affordability. There are opportunities to expand the scope of home care services and improve the quality of care. Policymakers may consider providing more resources and incentives to enhance HBMC in China. Educational programs may be created to train more HBMC providers and improve their professional skills.

